# Supplementary material for: MOCCASIN: a method for correcting for known and unknown confounders in RNA splicing analysis
Source: Nat Commun. 2021 Jun 7;12:3353. doi: 10.1038/s41467-021-23608-9 (PMC8184769; doi:10.1038/s41467-021-23608-9)
Supplement: Supplementary file 1 — Supplementary Information [file 41467_2021_23608_MOESM1_ESM.pdf]

# MOCCASIN: A method for correcting for known and unknown confounders in RNA splicing analysis

Slaff et al.

# 1 Supplementary Information

## 1.1 RNA-Sequencing data processing steps

SRA files were converted to fastqs with fastq-dump (sratoolkit.2.9.2) using the following commands: --split-3 –gzip. Sequencing adaptors and low quality base calls were trimmed from fastqs using trim_galore (0.5.0) using the following commands: --stringency 5 --length 35 -q 20. For gene expression analyses, transcript per million (TPMs) and effective length quantifications were obtained using Salmon (0.11.3) in mapping-based mode with default settings. The Salmon transcriptome indices were prepared with the GRCh38 annotation for human samples and the Gencode M21 annotation for mouse samples. For alternative splicing analyses, trimmed fastqs were aligned with STAR (2.5.2a) using the following commands: --outSAMattributes All --alignSJoverhangMin 8 --readFilesCommand zcat --outSAMunmapped Within --outSAMtype BAM Unsorted. The aligned bams were sorted with samtools (1.9). The sorted bam files were then quantified for alternative splicing with MAJIQ (See 'Splicing analysis').

## 1.2 Toy data

The toy data was constructed to quantify the inaccuracies incurred by various models in a setting where a bias term causes a known change in % TPM (i.e. a change that matches the synthetic batch data generation process described above) such that the more used junction in a two-way LSV changes by that % TPM while the other junction increases by the same amount of TPM to maintain overall expression.

Given the above data we follow the MOCCASIN correction procedure for batch effects: First, we scale the junction reads so the LSV has the same coverage over all samples. Then we apply the transformation using numpy (identity, arcsinh, log), then fit the linear model and perform the read rates adjustments accordingly, then apply the inverse transformation, then compute PSI from the adjusted reads. The results of this analysis are plotted with matplotlib in Supplementary Figure S7 (see the Supplementary Files for the code to reproduce the toy data).

## 1.3 TARGET dataset

The TARGET data used in this paper comprises 579 patients, from which samples were taken either at primary leukemia diagnosis or after relapse. Most of the patients' samples were sequenced in 2-3 technical duplicates, and as indicated in the sequencing meta data, samples were sequenced either on an Illumina HiSeq 2000 or 2500. There was also information about sample release and load date, but these dates were completely confounded with our biological variable of interest (primary diagnosis vs relapse). We observed that the samples deriving from the Illumina HiSeq 2000 or 2500 exhibited batch effects (Fig1a-b). It is possible that the HiSeq 2000 and 2500 have distinct biases, or it is possible that the sequencer label is a proxy for how the samples were handled. For example, samples may have been frozen for a longer period of time prior to HiSeq 2500 vs 2000 sequencing. Or, perhaps different technicians processed the 2500 vs 2000 samples.

Without further information about how the samples were processed, handled, stored, etc., it is difficult to determine the cause of 2000 vs 2500 sample batch effects we observed.

The clinical information associated with the 579 TARGET patients indicates their leukemias break down as follows: 160 with a B cell of origin, 265 with a T cell of origin, and 154 with an unknown cell of origin. We predicted the 154 patients' leukemia cells of origin using UMAP of splicing (PSI) and gene expression (TPM). The UMAP analysis is identical to Figure 1a-b, but colored by cell of origin. Of the 154 patients with an unknown cell of origin, 90 cluster with B cell of origin patients and 64 cluster with T cell of origin patients. Thus, we predicted that 250 leukemias have a B cell of origin and 329 leukemias have a T cell of origin.

## 1.4 ENCODE dataset

The ENCODE RNA-Sequencing data used in this paper were described in a preprint (Van Nostrand et al. 2020) which used 1100 ENCODE RNA-Sequencing samples generated in 61 distinct experiments (batches), whereby each batch had a control and at least one knockdown target. Every ENCODE sample, including the controls, had two biological replicates performed in every batch. 29 (548 samples) and 32 (552 samples) batches used HepG2 cells or K562 cells, respectively.

Van Nostrand et al. reported they identified batch effects in their data but did not quantify those. To correct for those, Van Nostrand et al applied the ComBat batch correction algorithm directly on sample read counts, and then averaged together the controls across batches to create two "virtual" control replicates before quantifying differential expression and differential splicing.

Similar to Van Nostrand et al, in this work HepG2 cells and K562 cells were considered separately when modelling and quantifying batch effects. However, given the ENCODE experimental design described above it was not clear what would be the optimal approach to model batch effects. After testing a variety of strategies and measuring their effects using the metrics described in this paper we converged to the following procedure.

First, batch effects in HepG2 and K562 samples were modelled and removed independently by MOCCASIN. After batch-correction, controls across all batches were then considered altogether in one group when quantifying differential splicing between control and knockdowns, separately for HepG2 and K562 samples. Differential splicing was quantified with MAJIQ (see 'Splicing analysis' for more details). For differential expression analysis, control replicate 1 samples and control replicate 2 samples' gene level TPMs across all the batches were averaged together to create two "virtual" control replicates.

Overall, there were 1100 control vs target comparisons included in the analysis presented in the various plots and heatmaps presented here. Specifically, for the heatmaps and clustering figures LSVs were filtered to select at most one representative junction such that at least one knockdown to controls comparison had a |dPSI|>=0.1 and Wilcoxon two-sided test p-value less than 0.05. Then for each LSV, the junction with the greatest |dPSI| value kept, breaking ties randomly.

## 1.5 Peikoto et al dataset and analyzing batch effect associated with different laboratories

RNA-Seq of mouse hippocampus from (Peixoto et al. 2015) were downloaded with fastq-dump v2.9.2 from GEO (Wood lab, GSE44229; Abel lab, GSE63412). Reads were trimmed with trimgalore v0.4.4_dev, aligned to Gencode M20 using STAR v2.6.1a, and analyzed for splicing with MAJIQ v2.1-46f7268. Samples SRRs processed included: SRR7072**19-24**, 6 samples, Wood lab Object-Location Memory (OLM) treatment; SRR7072**25-30**, 6 samples, Wood lab, control treatment, SRR16566**67-71**, 5 samples, Abel lab, control treatment; SRR16566**73-76**, 4 samples, Abel lab, Fear Conditioning (FC) treatment.

MOCCASIN model matrix included: intercept, Treatment_OLM, Treatment_FC. No explicit confounder factor was included even though we know the Lab_Wood / Lab_Abel difference. Instead, we used MOCCASIN -U (see methods above) with 2 hidden factors. For evaluation, 10,000 LSVs were selected uniformly at random from those originally quantified in all samples. Principal component analysis (PCA) from scikit-learn was applied to PSI from the 10,000 LSVs, and plots were generated with matplotlib. MAJIQ was used to quantify differences in splicing between Wood vs Abel control samples and Wilcoxon two sided rank test applied to quantify p-values.

See Supplementary Figure 1 for the results of this analysis.

## 1.6 Gene expression analysis

For gene expression level analysis of the TARGET and ENCODE datasets, transcript level TPM quantifications were collapsed into gene level TPMs with tximport (bioconductor-tximport 1.12.0). For differential expression analysis of ENCODE, DESeq2 was used to quantify the log2 fold-change (shrunk with DESeq2::fcShrink using the apelgm method) and statistical significance of differential expression between the two "virtual" control replicates and a given pair of knockdown target replicates. The DESeq2 adjusted p-value of 0.05 was the threshold for identifying a differentially expressed gene as statistically significant.

## 1.7 Splicing analysis

MAJIQ and VOILA (v2.1-46f7268) were used to quantify splicing from sorted bam files. MAJIQ uses a Bayesian model for both PSI and dPSI, computing expected and posterior probabilities over those. Thus, when point estimates for PSI or dPSI are described in the text they generally refer to the expected values for PSI or dPSI with a slight abuse of notation to improve readability: For example PSI > 0.1 instead of E[PSI] > 0.1, unless the posterior probability distribution is stated explicitly (e.g. P(|dPSI| > *C*)). For defining differentially spliced events two MAJIQ based methods were used. The first is MAJIQ's Bayesian expected delta PSI (dPSI) mentioned above and described in (Vaquero-Garcia et al. 2016), which is geared to produce a set of high confidence splicing changes between groups of biological replicates. Briefly, MAJIQ dPSI quantifies the probability that a difference in splice inclusion is above some threshold with a certain confidence i.e. (P(|dPSI| >= *C1*) >= *C2*) for example . We used a threshold of *C1* = 10% or 20% in the results

reported in the main and supplementary figures. Significant differences in splicing were those identified as having a *C2* = 95% probability of being greater than the given *C1* threshold.

The second method to detect differential splicing involved either a Student's t test or a Wilcoxon two sided rank test combined with an optional threshold on the observed difference between the median over the expected PSI of samples from the two groups. This approach was aimed to accommodate heterogeneous groups, as we observed in ENCODE's CTRL samples (see description in ENCODE data section).

## 1.8 Uniform manifold approximation and projection (UMAP)

UMAP (McInnes, Healy, and Melville 2018) was applied to gene expression (TPM and log2 fold-change) data and splicing (PSI, dPSI, and difference in median PSI) data using the R package umap (0.2.4.1) with method set to 'umap-learn', n_neighbors set to 25, and the random seed set to 924319452. UMAP projections were visualized with the R packages extrafont, ggplot2, and patchwork.

## 1.9 Calculating percent variance explained by confounders

Percent variance explained by a confounder was calculated by the R^2 when fitting a linear model with batch effect as a covariate.

## 1.10 Hierarchical clustering

For hierarchical clustering of gene expression (TPM and log2 fold-change) data and splicing (PSI, dPSI, and difference in median PSI) data, the ward.D2 algorithm was applied to Euclidean distances between columns and between rows. Clustering results were visualized with the R packages extrafont, ComplexHeatmap, and Pheatmap.

## 1.11 Scatterplots and Venn Diagrams

Scatterplots with lines of best fit and Pearson correlations were generated using the base R lm() method. Size-scaled Venn Diagrams were generated with R package eulerr (6.1.0).

## 1.12 Assessing MOCCASIN effect on differentially spliced events

LSVs were called changing if they met the thresholds (e.g. dPSI>=0.1) as specified for a given analysis and matching plot in this work. Note that in this work we only aimed to assess MOCCASIN's correction effect. Thus, to avoid introducing other variables into the analysis we used the same algorithm (MAJIQ) and the same settings as ground truth when these were applied to the original simulated data without batch effects introduced. This comparison between the analysis of the original data, perturbed data, and corrected data gave the true negative (TN), true positive (TP), false positive (FP), and false negative (FN) estimates and their associated statistics (FPR, FDR) used in the main text and figures.

## 1.13 Modeling unknown confounding factors

MOCCASIN's model matrix input by the user includes filenames, variables of interest, and known confounders. The user may optionally request that MOCCASIN identify additional factors of unwanted variation, by setting the -U option to 1 or more.

In order to discover factors of variation in the data which are not explained by the factors in the model matrix, MOCCASIN first computes for each LSV, the junction with the highest variance in PSI over samples. Then, the top NUM_JUNCS (default: 10,000) such junctions by variance are selected as the input data $\mathbf{Y}$ for the following procedure:

1. Perform OLS regression on the input data $\mathbf{Y}$ with this model matrix.
2. Compute the residuals $\mathbf{Y_r}$ from this regression.
3. Compute the singular value decomposition: $\mathbf{WSV}^{T}=\mathbf{Y_r}$
4. Append the estimated factors W (up to the number specified by the -U argument) to the original model matrix and retain them if the model remains full-rank.

In a nutshell, the above method for discovering unknown confounding factors is a variant of matrix decomposition as are many of the previously published works on correcting confounders for expression data. Specifically, the application of SVD on the most highly varying features was previously suggested by Risso et al and included in the RUV package.

Compared to RUV, we made the following modifications: First, the RUVs procedure assumes that only two kinds of effects exist, termed "main variables of interest" and "unwanted variation"; it does not handle known confounders, and as currently implemented would rediscover known confounders corresponding to a large proportion of the variation in the data. Hence, our input to the SVD step used by RUV are the residuals $\mathbf{Y_r}$ .We note that these residuals may be negative. This means we were not able to simply apply RUV as a second processing step: RUVs expects counts and tries to take their logarithm and was therefore unworkable for our purposes.

In addition, as discussed in the main text we utilize OLS regression over read rates instead of a negative binomial GLM suggested by the RUV documentation. We also note that, like the RUVr procedure as implemented currently in RUVSeq (July 2019), our derived factors correspond to only the left singular vectors in the singular value decomposition; this implementation contrasts with the method reported in the 2014 Nat Biotech paper, which states that the derived factors should be the left singular vectors composed with the singular values.

## 1.14 Speed and memory optimizations

In contrast to gene or transcript expression studies, which typically utilize tens or at most a few hundred thousand tags, MOCCASIN must model and adjust counts for possibly millions of splice junctions in a single run. The need to handle that many inference tasks motivated MOCCASIN's simple model performing linear regression on junction read rates. However, the size of the data necessitates additional speed and memory optimizations.

MOCCASIN partitions LSVs into "chunks" for processing in order to limit peak memory usage. This is possible because LSVs are modeled and adjusted independently of one another in the MOCCASIN framework. The user can set the max chunk size in terms of the max number of

coverage table cells to be loaded at once into memory (-M). However, the peak memory use currently is lower-bounded by the size of the coverage table in the largest input .majiq file.

MOCCASIN allows the user to specify the number of processes to use (-J) as a speed optimization. This governs the number of spawned processes which will process each chunk of LSVs in parallel during the modeling and adjustment steps.

In practice, when running MOCCASIN on our simulated datasets we found MOCCASIN (-J 8) took 13 seconds to run on 4 samples and 34 seconds to run on 16 samples, with roughly linear increase in time, and used less than 1G of RAM (Supplementary Fig 8).

For large (i.e. >500 samples) datasets, I/O becomes a rate-limiting process since we try to keep memory use low (see above) by writing and reading temporary files to a user-specified tmp location. The temporary files would not be necessary if we made use of shared memory across processes. Python3.8 does include shared memory capabilities but python3.8 is not yet as widely used as python3.6 and python 3.6 lacks shared memory capabilities. To facilitate MOCCASIN usability, we chose to use python3.6, and to make up for lack of shared memory in python3.6, we suggest users point the MOCCASIN tmp directory to the fastest disk available (e.g. a RAM drive at /dev/shm). At the end of the MOCCASIN execution, the tmp subdirectory is automatically deleted (unless the -K flag is supplied). For example, on the TARGET dataset (885 samples), using a RAM drive and -J 40, MOCCASIN took 2.8 days to finish and used a maximum of 26G of RAM. We note that especially for large datasets the main bottleneck for execution time is I/O and not the algorithm itself.

# 2 Supplementary Figures

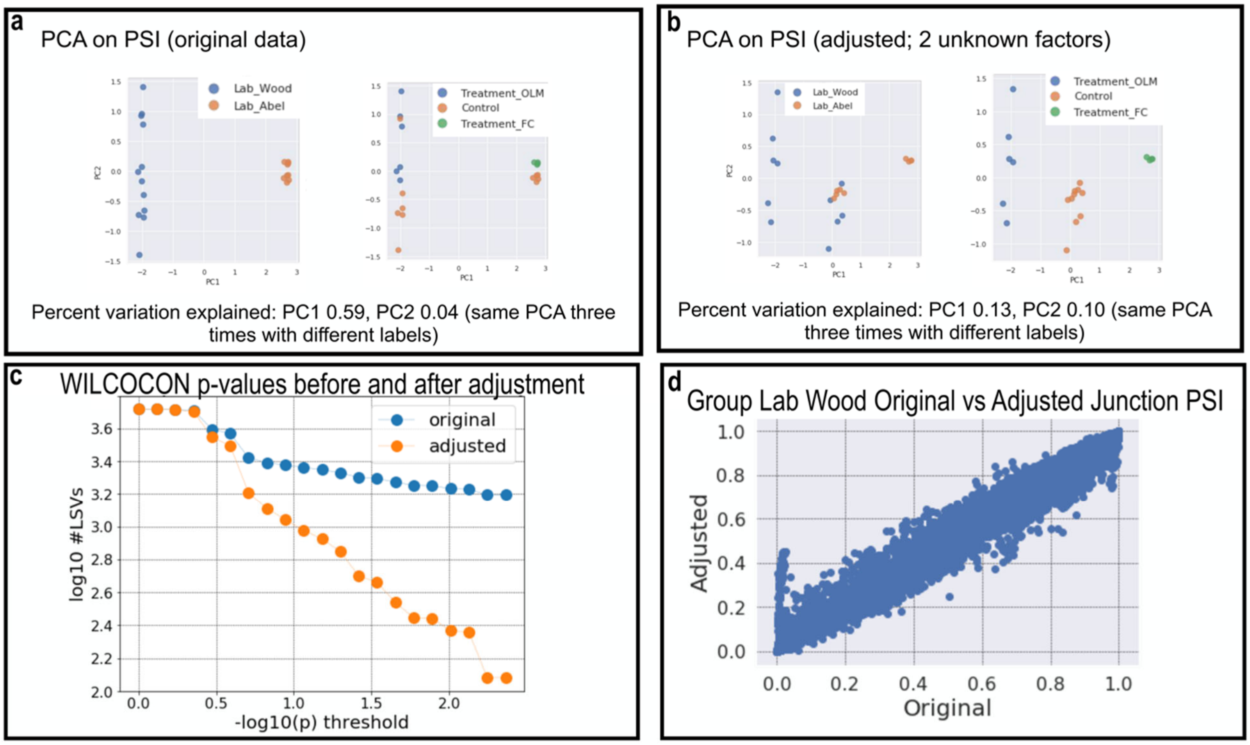


## 2.1 Supplementary Figure 1: MOCCASIN removes batch effects associated with different laboratories

PCA of PSI from 10,000 LSVs shows samples cluster by batch before MOCCASIN in the leftmost plot of **a** (12 Lab Wood batch samples in blue and 9 Abel batch samples in orange)**.** The right plot in **a** shows the same PCA colored by treatment (11 controls in orange, 6 OLM in blue, and 4 FC in green). After batch-correcting the data with MOCCASIN modeling for 2 hidden factors (-U 2, see methods), as shown in **b**, the samples cluster by treatment (same colors as in **a**). **c**, the number of LSVs (Y-axis) detected as differential (|E(dPSI)|>0.2) between Wood (N=6) versus Abel (N=4) control samples before (blue) vs after (orange) MOCCASIN across a range of increasingly significant p-values (X-axis, two-sided Wilcoxon rank sum test, $-\log_{10}$ scale). **d**, PSI of 10,000 LSVs from Wood (N=6) batch control samples before MOCCASIN (X-axis, original) vs Abel (N=4) batch control samples after MOCCASIN (Y-axis, adjusted). OLM, Object-oriented learning; FC, Fear Conditioning.

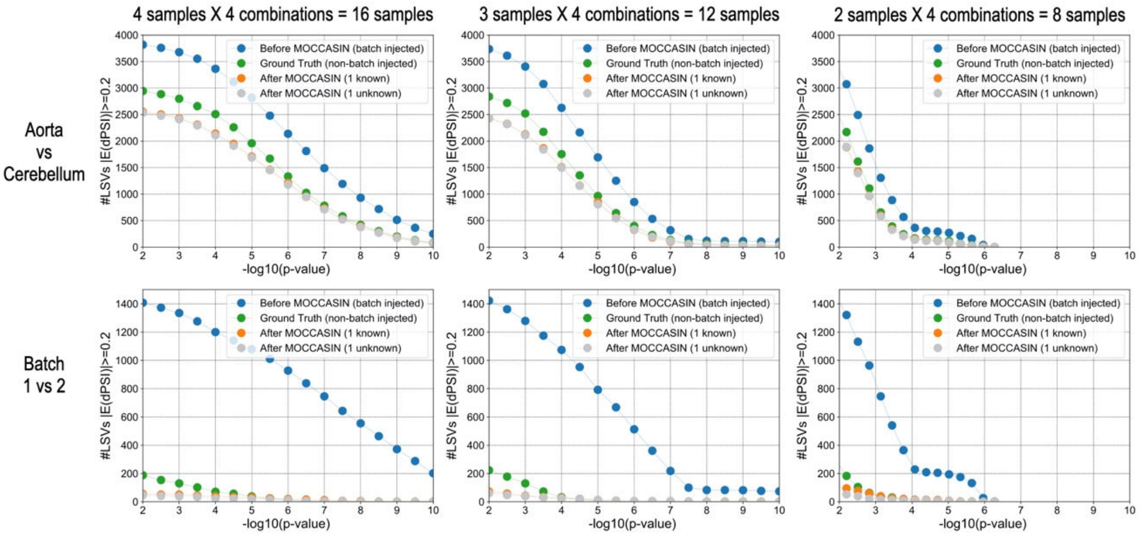


## 2.2 Supplementary Figure 2: MOCCASIN removes batch effects from experimental designs of varying size

This analysis repeats Figure 2b-c, but with decreasing sample sizes. Shown are the number of LSVs (Y-axis) detected as differential (|E(dPSI)|>0.2) for the aorta vs cerebellum signal (top row) and the batch 1 versus batch 2 signal (bottom row) across a range of increasingly significant p-values (X-axis, Student's t test, -$\log_{10}$ scale). Total number of samples used for each plot were 4, 3, or 2 times the number of tissue/batch combinations (2 tissues x 2 batches = 4). The green points ("Ground Truth") are from the simulated data with no with batch signal injection and the blue points ("Before MOCCASIN") are from the same data after batch signal injection (*G*=20%, *C*=60%). Both blue and green points serve as reference points for MOCCASIN correction of the batch signal and blue (no batch signal injection) points are from the simulated data without MOCCASIN correction and serve as a reference. Orange and grey represent respectively the results after MOCCASIN correction when the batches are known or unknown.

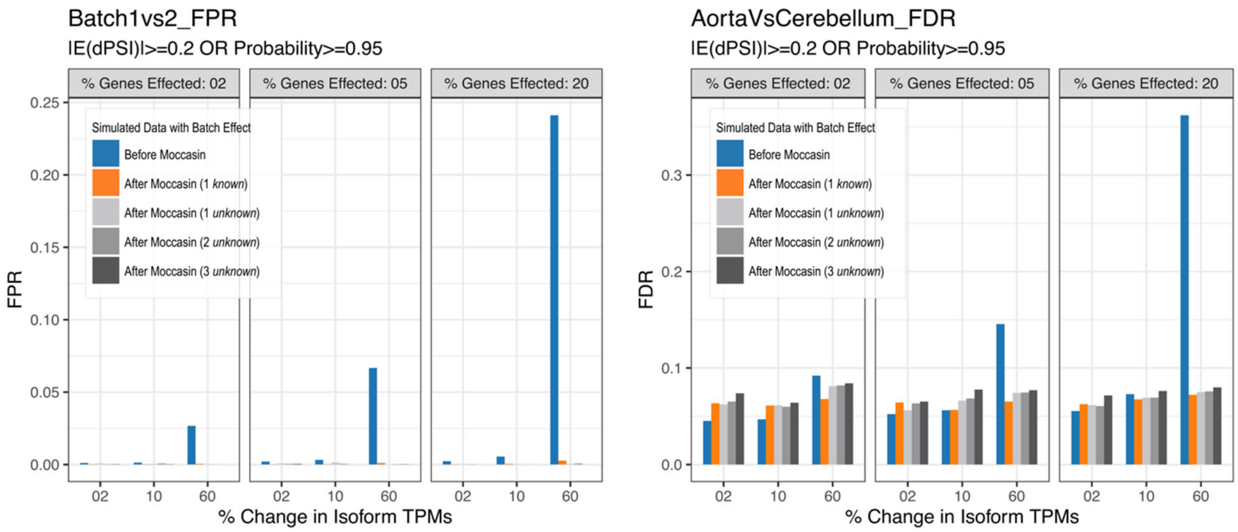


## 2.3 Supplementary Figure 3: Batch difference FPR and tissue difference FDR with MOCCASIN discovering 1-3 unknown confounders

This figure is related to figure 2b-e in the main text, where we used MAJIQ dPSI to quantify batch differences (batch 1 vs 2) and tissue differences (aorta vs cerebellum) from the simulated data. Recall that LSVs were called changing if they met the threshold Probability(|dPSI|>=0.2)>=0.95. See Section 1.12 above regarding how false positive rate (FPR) and false discovery rate (FDR) were calculated. Shown here on the left are Batch 1 (N=4) vs batch 2 (N=4) FPR and on the right aorta (N=4) vs cerebellum (N=4) FDR from the batch-effected simulated data (blue, before MOCCASIN) versus the corrected data (orange or grey, after MOCCASIN). Included in those plots are MOCCASIN runs in three different configurations: 1 known confounding factor provided to MOCCASIN (orange), or 1, 2, or 3 (gradient of light-to-dark greys) hidden confounding factors identified and removed by MOCCASIN. FPR, false positive rate; FDR, false discovery rate; TPM, transcripts per million; dPSI, delta percent splice included.

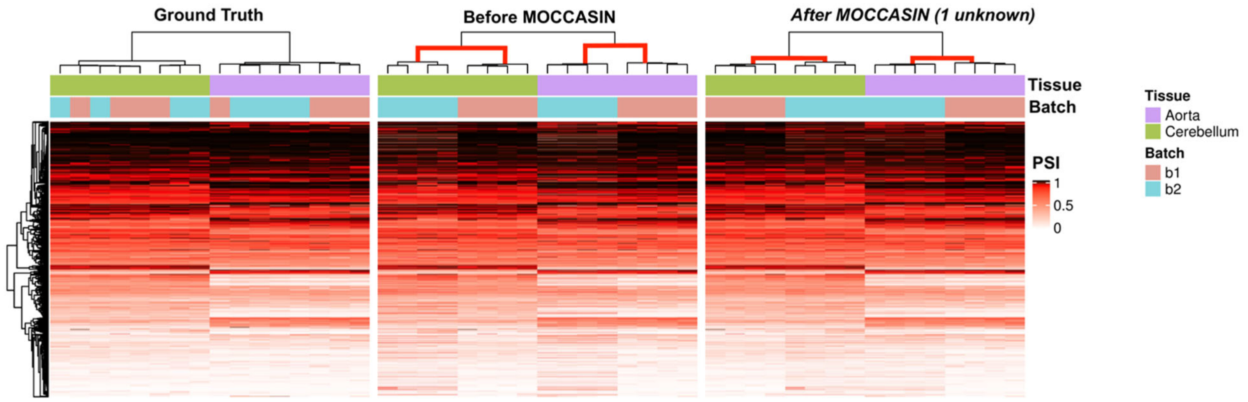


## 2.4 Supplementary Figure 4: Unsupervised clustering of splicing simulated data with batch effect when using MOCCASIN to discover an unknown confounder

This figure is related to figure 2e in the main text where the same analysis is shown but when using a known confounder to specify the batches. Heatmaps of PSI from simulated data without batch effect (ground truth, left), with simulated batch effect (G=20%, C=60%) without correction (middle), and after applying MOCCASIN with 1 unknown confounding factor (right). Each column is a sample (N=16), and each row is an LSV (N=7368). The colored bars above the samples denote the sample's tissue (8 aorta samples in purple, 8 cerebellum samples in green) and (8 batch 1 samples in red, 8 batch 2 samples in blue). On top, the dendrogram lines represents the Euclidean distances. Dendrogram lines are colored red to highlight the Euclidean distance between batches is greater before MOCCASIN than after MOCCASIN.

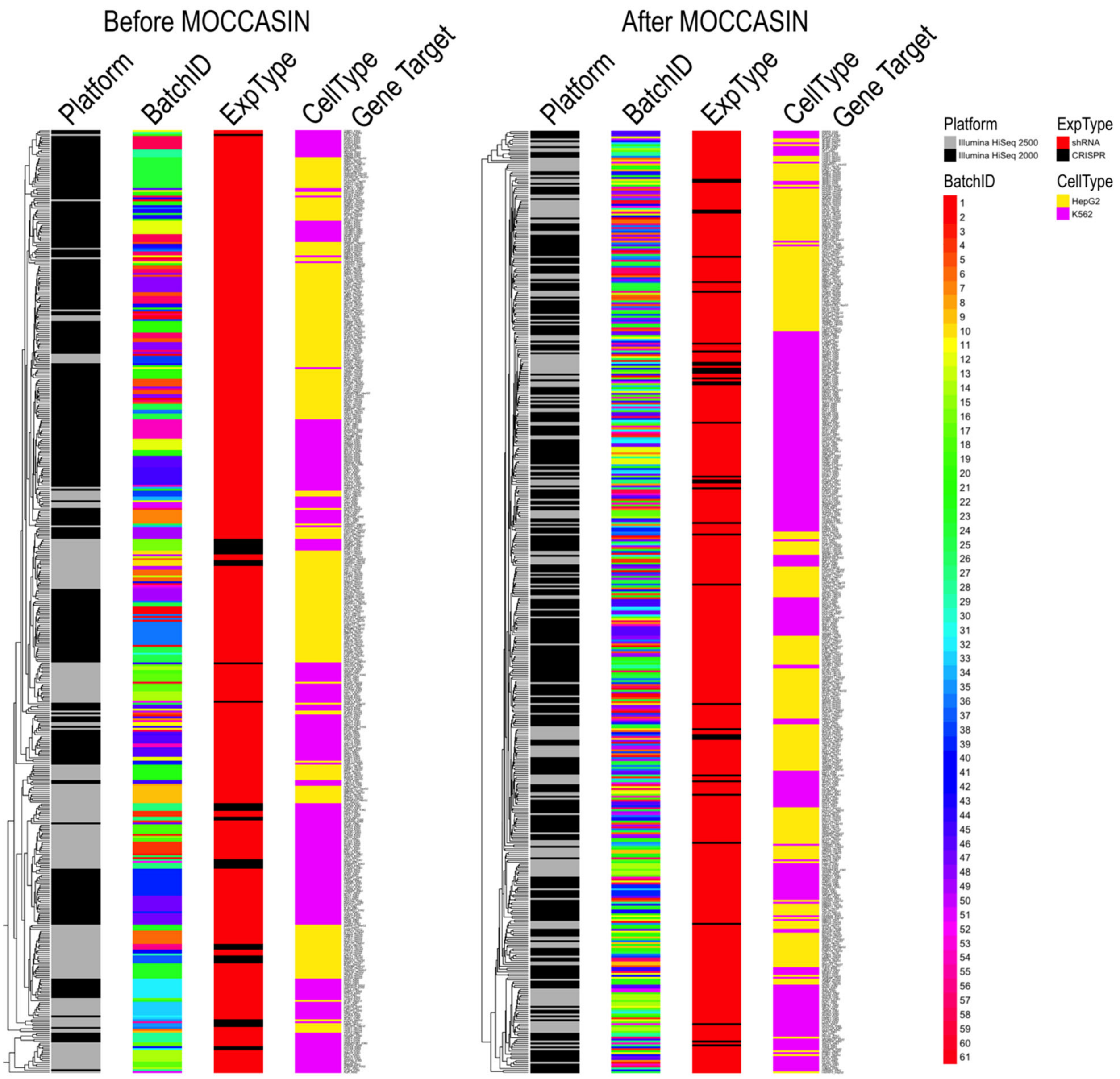


## 2.5 Supplementary Figure 5: Clustering of ENCODE dPSI before vs after MOCCASIN

This supplementary figure is related to figure 3a in the main text. Shown here are clustergrams of dPSI from 1100 ENCODE control vs target KD comparisons and 43081 LSVs. These LSVs were those exhibiting a splicing change with a Wilcoxon two-sided test P-value less than 0.05 and |dPSI|>0.1 in at least one comparison. Each comparison consists of 2 KD experiments of one target versus 58 HepG2 or 64 K562 matched cell type control samples. Clustergram bars on the left indicate Euclidean distances between KD experiments. Each colored bar provides information about the given experiment: the sequencing platform (HiSeq 2000, black; HiSeq 2500, grey), the batch identifier (1:61, rainbow-colored), the type of KD experiment (shRNA, black; CRISPR knockout, red), and the cell type (HepG2, yellow; K562, pink). The left figure is before

MOCCASIN, and right is after MOCCASIN. Note that after MOCCASIN, Batch IDs colors no longer segregate together, rather, the colors are dispersed randomly. KD, knockdown.

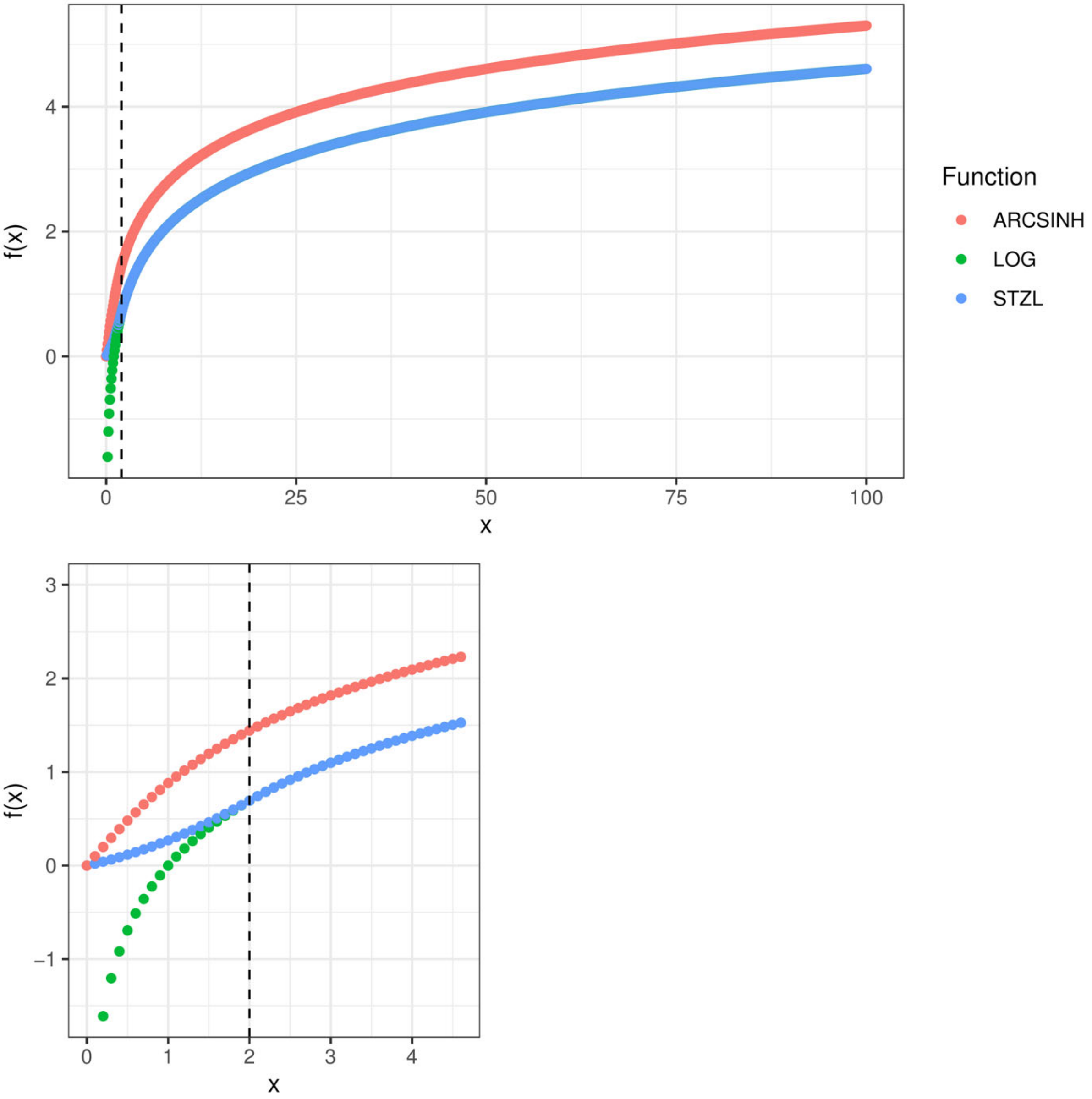


## 2.6 Supplementary Figure 6: The smoothed toward zero log (STZL) function

Illustration of the STZL (blue) function compared to Log (green) and Asinh (red). Top, evaluation of the functions from 0 < x <= 100. Bottom, evaluation of the functions from 0 < x <= 10. Dotted line drawn at x=2. STZL, smoothed toward zero log; ASINH, inverse hyperbolic sine.

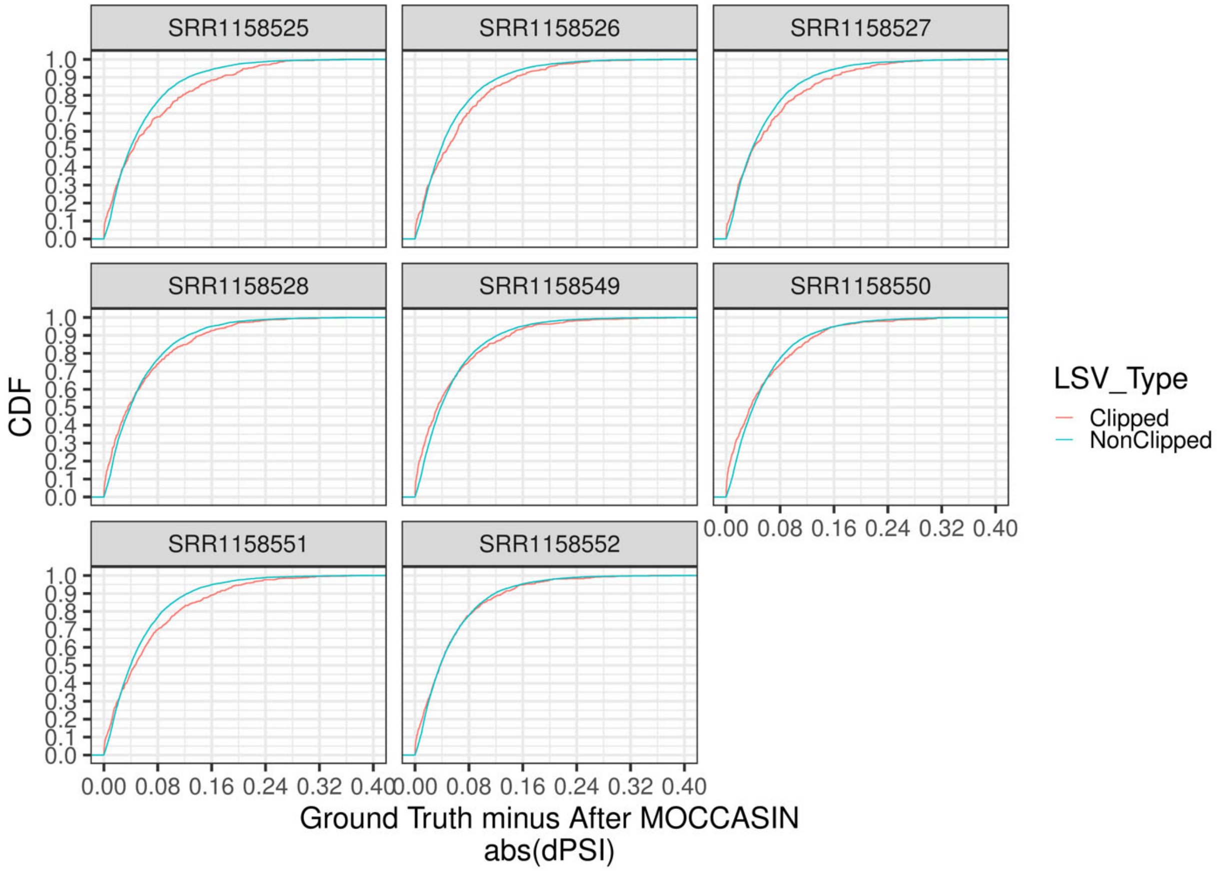


## 2.7 Supplementary Figure 7: Comparing MOCCASIN model accuracy for clipped and non-clipped LSVs

A CDF of the absolute difference in PSI (X-axis) between ground truth (PSI computed by MAJIQ over unperturbed samples, see Section 1.5 above) and PSI computed by MAJIQ after MOCCASIN correction. Results shown here are from all samples used with the simulated batch effect set to be the strongest (*G*=20%, *C*=60%, see main text). The plots includes only those LSVs that exhibited a batch-affected, defined as ([PSI Ground Truth] - [PSI After Batch Perturbation]) > 0.01. For each such batch-effected LSV, the most batch-effected junction was selected to represent that LSV. An LSV is identified as “clipped” if more than half of the 30 bootstraps from one junction were clipped by MOCCASIN. An LSV is identified as “non-clipped” if no bootstraps from any junction in the LSV were clipped. The red line shows accuracy of MOCCASIN on clipped and the green line shows MOCCASIN accuracy for the majority of the LSVs which were not clipped. Overall, we find across all samples 503-607 LSVs are clipped compared to 6048-7133 which are not, i.e. only ~7% are clipped. The relation between the green and red line vary between sample, with a possible small advantage in overall accuracy for the non-clipped (green) line. In the most extreme case (sample SRR 1158525) we find that to achieve the same fraction of events (y-axis at 0.8) “costs” an increase of accepted dPSI (x-axis) by 0.04.

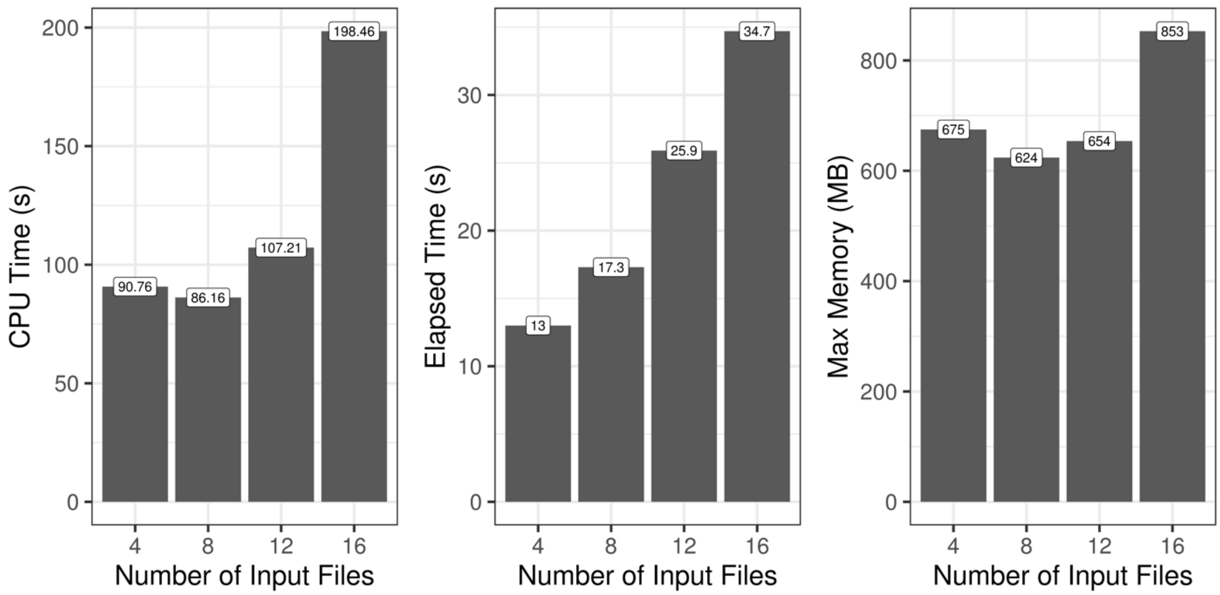


## 2.8 Supplementary Figure 8: Runtime and memory usage of MOCCASIN

Computational cost of MOCCASIN run with 8 threads was evaluated with 4, 8, 12, or 16 input files (simulated data, see main text methods) on a Dell C6420 Quad node system with 100 GB/s FDR InfiniBand connection to an HPC filesystem.

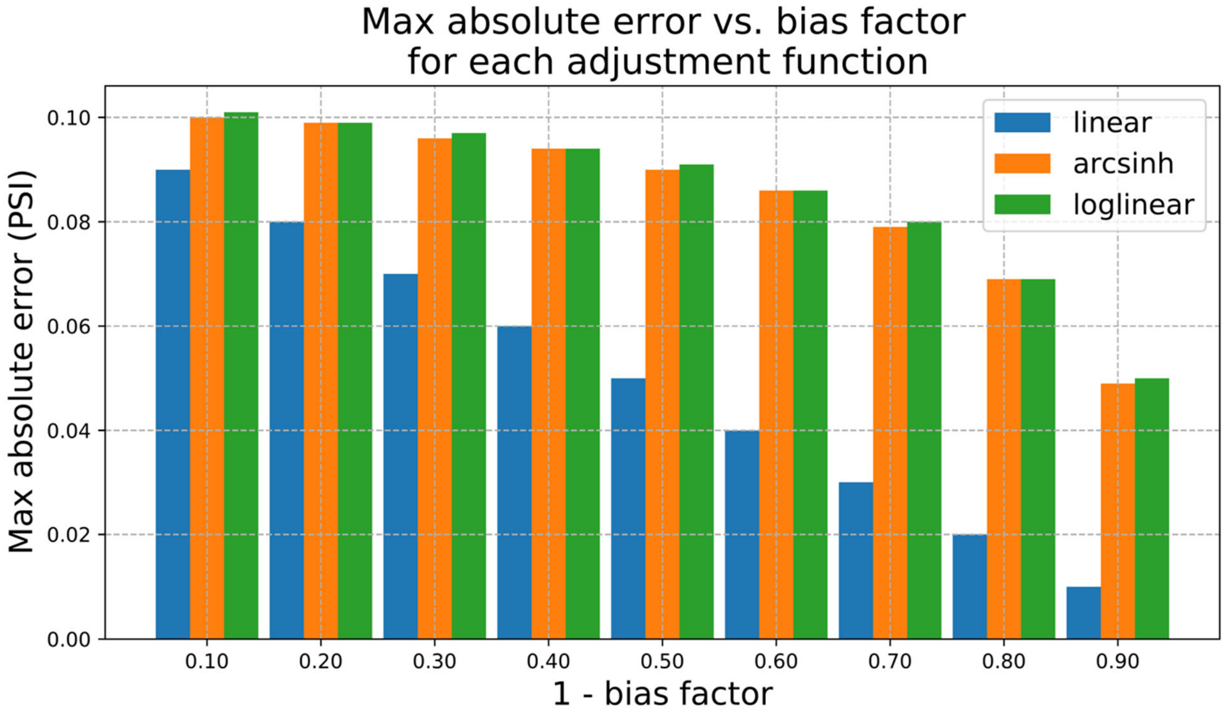

## 2.9 Supplementary Figure 9: MOCCASIN correction inaccuracies for toy data with a multiplicative bias factor

The procedure to create the toy data shown in the figure is described in Section 1.2. The 1-bias factor (x-axis) varies from 0.9 which corresponds to a 10% change in TPM for the more used LSV junction, to 0.1 which corresponds to a 90% change in TPM. Inaccuracies incurred by the MOCCASIN bias correction procedure are shown for linear read rates (blue), arcsinh transformed read rates (orange) and a log linear model (green). Notice that for significant batch effects of 20% a maximum of 2% dPSI inaccuracy is observed for the linear model compared to 7% by the other two models. Even for severe switch effects of 60% change in TPM which were modeled in the toy data, a maximum of 6% dPSI inaccuracy is observed for the linear model compared to ~9.5% by the other two models.

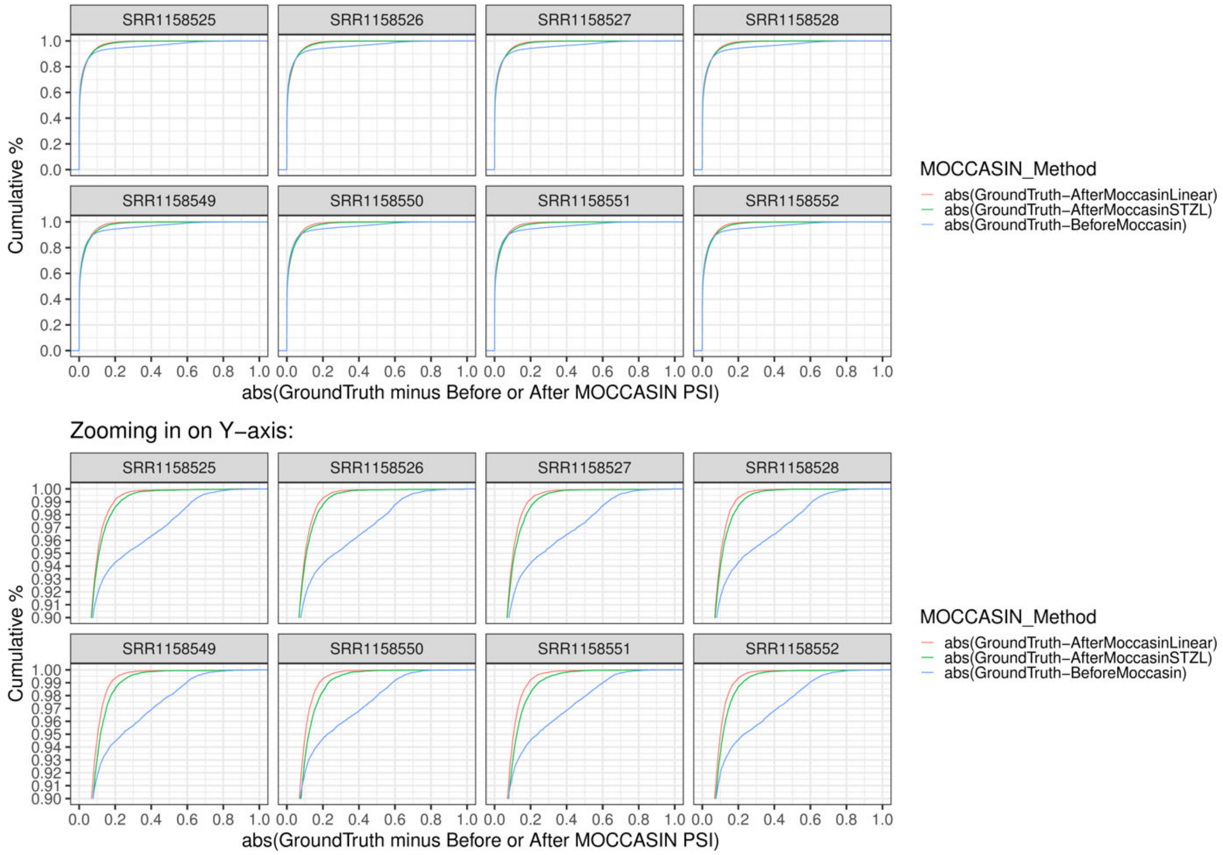


## 2.10 Supplementary Figure 10: Comparing performance of MOCCASIN linear and STZL models on synthetic data

Shown are CDF of samples' absolute differences in PSI (X-axis) between ground truth minus the batch effect (blue line, *G*=20%, *C*=60%), or after the sample was corrected by MOCCASIN with the default linear model (red line) or the optional STZL model (green line). In other words, the CDF shows the effectiveness of MOCCASIN to correct PSI back towards ground truth whereby a smaller difference in PSI can be interpreted as a more effective correction toward ground truth.

The top plots show the full scope of the CDF (Y-axis from 0 to 1) and the bottom plots are a subset of the top CDFs, highlighting performance of MOCCASIN on the top 10% of the most batch effected LSVs (out of a total of 21566).

# 3 Supplementary Files

In order to facilitate reproducibility of our analysis and supply figures that capture additional results we created several dedicated .zip files, the content of each is described in the following Zenodo repository: http://doi.org/10.5281/zenodo.4294189.

# 4 Supplementary References

Peixoto, Lucia, Davide Risso, Shane G. Poplawski, Mathieu E. Wimmer, Terence P. Speed, Marcelo A. Wood, and Ted Abel. 2015. "How Data Analysis Affects Power, Reproducibility and Biological Insight of RNA-Seq Studies in Complex Datasets." *Nucleic Acids Research* 43 (16): 7664–74. https://doi.org/10.1093/nar/gkv736.

Van Nostrand, Eric L., Peter Freese, Gabriel A. Pratt, Xiaofeng Wang, Xintao Wei, Rui Xiao, Steven M. Blue, et al. 2020. "A Large-Scale Binding and Functional Map of Human RNA-Binding Proteins." *Nature* 583 (7818): 711–19. https://doi.org/10.1038/s41586-020-2077-3.

Vaquero-Garcia, Jorge, Alejandro Barrera, Matthew R. Gazzara, Juan González-Vallinas, Nicholas F. Lahens, John B. Hogenesch, Kristen W. Lynch, and Yoseph Barash. 2016. "A New View of Transcriptome Complexity and Regulation through the Lens of Local Splicing Variations." *ELife* 5 (February): e11752. https://doi.org/10.7554/eLife.11752.
