# Supplementary material for: MOCCASIN: a method for correcting for known and unknown confounders in RNA splicing analysis
Source: Nat Commun. 2021 Jun 7;12:3353. doi: 10.1038/s41467-021-23608-9 (PMC8184769; doi:10.1038/s41467-021-23608-9)
Supplement: Supplementary file 3 — Reporting Summary [file 41467_2021_23608_MOESM3_ESM.pdf]

nature research

Corresponding author(s): Yoseph Barash

Last updated by author(s): Mar 29, 2021

Reporting Summary

Nature Research wishes to improve the reproducibility of the work that we publish. This form provides structure for consistency and transparency in reporting. For further information on Nature Research policies, see our Editorial Policies and the Editorial Policy Checklist.

Statistics

For all statistical analyses, confirm that the following items are present in the figure legend, table legend, main text, or Methods section.

n/a	Confirmed	
☐	☒	The exact sample size (*n*) for each experimental group/condition, given as a discrete number and unit of measurement
☐	☒	A statement on whether measurements were taken from distinct samples or whether the same sample was measured repeatedly
☐	☒	The statistical test(s) used AND whether they are one- or two-sided<br>*Only common tests should be described solely by name; describe more complex techniques in the Methods section.*
☐	☒	A description of all covariates tested
☐	☒	A description of any assumptions or corrections, such as tests of normality and adjustment for multiple comparisons
☐	☒	A full description of the statistical parameters including central tendency (e.g. means) or other basic estimates (e.g. regression coefficient) AND variation (e.g. standard deviation) or associated estimates of uncertainty (e.g. confidence intervals)
☒	☐	For null hypothesis testing, the test statistic (e.g. *F*, *t*, *r*) with confidence intervals, effect sizes, degrees of freedom and *P* value noted<br>*Give P values as exact values whenever suitable.*
☒	☐	For Bayesian analysis, information on the choice of priors and Markov chain Monte Carlo settings
☒	☐	For hierarchical and complex designs, identification of the appropriate level for tests and full reporting of outcomes
☐	☒	Estimates of effect sizes (e.g. Cohen's *d*, Pearson's *r*), indicating how they were calculated

Our web collection on statistics for biologists contains articles on many of the points above.

Software and code

Policy information about availability of computer code

Data collection: BEERS algorithm (http://cbil.upenn.edu/BEERS/) to generate simulated RNA-Seq data

Data analysis:

```
 - r-argparse
 - r-tidyverse
 - r-ggplot2
 - r-pheatmap
 - r-upsetr
 # for some reason, eulerr needs this particular openblas version:
 - conda-forge::openblas==0.3.8
 - r-eulerr==6.1.0
 - r-umap==0.2.4.1
 - umap-learn==0.4.1 # python package used by r-umap under the hood to do umap maths
 - bioconductor-complexheatmap # used for clustergram w/ heatmap (I know I also have pheatmap...)
 - r-circlize # used for colors in the heatmap
 - r-extrafont # provides Arial font used by cowplots
# need to run this once in an interactive R session:
# library(extrafont); font_import(); loadfonts()
# These python modules are for running the moccasin algorithm
 - pandas
 - numpy
 - scipy
 - seaborn
 - scikit-learn #sklearn
```

- 2.1-46f7268
- matplotlib
- jupyterlab
- xarray
- r-irkernel # for jupyter notebook integration of R

For manuscripts utilizing custom algorithms or software that are central to the research but not yet described in published literature, software must be made available to editors and reviewers. We strongly encourage code deposition in a community repository (e.g. GitHub). See the Nature Research guidelines for submitting code & software for further information.

Data

Policy information about availability of data

All manuscripts must include a data availability statement. This statement should provide the following information, where applicable:
- Accession codes, unique identifiers, or web links for publicly available datasets
- A list of figures that have associated raw data
- A description of any restrictions on data availability

The TARGET results published here are in whole or part based upon data generated by the Therapeutically Applicable Research to Generate Effective Treatments (https://ocg.cancer.gov/programs/target) initiative, phs000218. The TARGET data used for this analysis were accessed under Project #10088: Alternative splicing in pediatric cancers (request 41466-5). The SRA toolkit was used to download sra files for the TARGET dataset and simulated data was based off of data from (Zhang et al. 2014) (GEO accession GSE54652). The ENCODE project fastqs were downloaded from www.encodeproject.org (See Supplementary Table 1 for a list of file accessions used). Data and scripts for replication available at https://bitbucket.org/cradens/moccasin_paper/ http://doi.org/10.5281/zenodo.4294189

Field-specific reporting

Please select the one below that is the best fit for your research. If you are not sure, read the appropriate sections before making your selection.

☒ Life sciences ☐ Behavioural & social sciences ☐ Ecological, evolutionary & environmental sciences

For a reference copy of the document with all sections, see nature.com/documents/nr-reporting-summary-flat.pdf

Life sciences study design

All studies must disclose on these points even when the disclosure is negative.

Sample size	All data derive from publicly available datasets; we used all available samples from each given dataset.
Data exclusions	No samples were excluded.
Replication	Data and associated scripts to replicate all analyses for the paper are available here: https://bitbucket.org/cradens/moccasin_paper/
Randomization	Not relevant. The purpose of this paper is to quantify batch/covariates effects and correct those using the method proposed in the paper.
Blinding	Blinding is not applicable because we generated the data ourselves or used previously generated data.

Reporting for specific materials, systems and methods

We require information from authors about some types of materials, experimental systems and methods used in many studies. Here, indicate whether each material, system or method listed is relevant to your study. If you are not sure if a list item applies to your research, read the appropriate section before selecting a response.

Materials & experimental systems

n/a	Involved in the study	
☒	☐	Antibodies
☒	☐	Eukaryotic cell lines
☒	☐	Palaeontology and archaeology
☒	☐	Animals and other organisms
☒	☐	Human research participants
☒	☐	Clinical data
☒	☐	Dual use research of concern

Methods

n/a	Involved in the study	
☒	☐	ChIP-seq
☒	☐	Flow cytometry
☒	☐	MRI-based neuroimaging
